# Genome and Environmental Activity of a *Chrysochromulina parva* Virus and Its Virophages

**DOI:** 10.3389/fmicb.2019.00703

**Published:** 2019-04-05

**Authors:** Joshua M. A. Stough, Natalya Yutin, Yuri V. Chaban, Mohammed Moniruzzaman, Eric R. Gann, Helena L. Pound, Morgan M. Steffen, Jenna N. Black, Eugene V. Koonin, Steven W. Wilhelm, Steven M. Short

**Affiliations:** ^1^Department of Microbiology, The University of Tennessee, Knoxville, Knoxville, TN, United States; ^2^National Center for Biotechnology Information, National Library of Medicine, National Institutes of Health, Bethesda, MD, United States; ^3^Department of Biology, University of Toronto Mississauga, Mississauga, ON, Canada; ^4^Department of Biology, James Madison University, Harrisonburg, VA, United States

**Keywords:** giant viruses, algae, NCLDV, freshwater, virophage, genome

## Abstract

Some giant viruses are ecological agents that are predicted to be involved in the top-down control of single-celled eukaryotic algae populations in aquatic ecosystems. Despite an increased interest in giant viruses since the discovery and characterization of *Mimivirus* and other viral giants, little is known about their physiology and ecology. In this study, we characterized the genome and functional potential of a giant virus that infects the freshwater haptophyte *Chrysochromulina parva*, originally isolated from Lake Ontario. This virus, CpV-BQ2, is a member of the nucleo-cytoplasmic large DNA virus (NCLDV) group and possesses a 437 kb genome encoding 503 ORFs with a GC content of 25%. Phylogenetic analyses of core NCLDV genes place CpV-BQ2 amongst the emerging group of algae-infecting Mimiviruses informally referred to as the “extended *Mimiviridae*,” making it the first virus of this group to be isolated from a freshwater ecosystem. During genome analyses, we also captured and described the genomes of three distinct virophages that co-occurred with CpV-BQ2 and likely exploit CpV for their own replication. These virophages belong to the polinton-like viruses (PLV) group and encompass 19–23 predicted genes, including all of the core PLV genes as well as several genes implicated in genome modifications. We used the CpV-BQ2 and virophage reference sequences to recruit reads from available environmental metatranscriptomic data to estimate their activity in fresh waters. We observed moderate recruitment of both virus and virophage transcripts in samples obtained during *Microcystis aeruginosa* blooms in Lake Erie and Lake Tai, China in 2013, with a spike in activity in one sample. Virophage transcript abundance for two of the three isolates strongly correlated with that of the CpV-BQ2. Together, the results highlight the importance of giant viruses in the environment and establish a foundation for future research on the physiology and ecology CpV-BQ2 as a model system for algal Mimivirus dynamics in freshwaters.

## Introduction

Viruses are the most abundant biological entities on Earth and play important roles in global ecosystems (Brussaard et al., 2008). Most of the currently described virus diversity is for particles smaller than 200 nm with genomes encoding the minimal functions necessary for replication, virion formation, and evasion of host defenses. As such, it was as a major surprise when the Mimiviruses were discovered infecting *Acanthamoeba* species: the size and complexity of these viruses rival those of many bacteria (Raoult et al., 2004; Wilhelm et al., 2017). Radically different from conventional model viruses, these giant viruses possess hundreds of genes, many of which are responsible for functions previously only found in cellular life, including substantial parts of translation machinery and auxiliary metabolic functions (Filee et al., 2008; Schulz et al., 2017; Schvarcz and Steward, 2018). The large particle and genome size of the giant viruses as well as the presence of genes that are universal among cellular life forms led to claims that these viruses blurred the traditional boundaries between cellular life and viruses, and even instigated a debate on possible origin of these viruses from an extinct fourth domain of cellular life as opposed to their evolution from smaller, simpler viruses (Moreira and Lopez-Garcia, 2005, 2009; Yutin et al., 2009; Claverie and Abergel, 2010; Colson et al., 2012; Yutin et al., 2014). Further fueling the discussion on the nature of giant viruses, the study of these viruses has also led to the discovery of virophages, small viruses that rely on a “host” giant virus’s protein machinery to replicate, usually at the expense of the giant virus (La Scola et al., 2008; Gaia et al., 2014). Virophage are distant relatives of polintons, eukaryotic virus-like self-synthesizing transposons, and polinton-like viruses (PLV), some of which apparently also parasitize giant viruses (Fischer and Suttle, 2011; Krupovic and Koonin, 2015; Yutin et al., 2015).

After careful examination, Mimiviruses as well as subsequently discovered giant viruses, such as Pandoraviruses and Pithoviruses, were shown to be physically larger members of a more diverse viral group referred to as the Nucleocytoplasmic Large DNA Viruses (NCLDVs) (Iyer et al., 2001, 2006; Koonin and Yutin, 2010), or the proposed order “Megavirales” (Colson et al., 2013). Despite the variety in the genome content and viral host ranges, the NCLDVs apparently share a common virus ancestor as indicated by the presence of 5 key universal genes along with about 35 additional genes that are mapped to the common ancestor by phylogenetic maximum likelihood reconstruction of gene gains and losses (Yutin et al., 2009; Koonin and Yutin, 2010). Furthermore, phylogenetic analysis of giant virus genes, particularly those that encode components of the translation system, suggests these genes were acquired from eukaryotic hosts on multiple occasions by different groups of the NCLDVs (Moreira and Brochier-Armanet, 2008; Yutin et al., 2014; Schulz et al., 2017). These findings suggest that giant viruses have evolved from smaller NCLDVs on multiple, independent occasions (Schulz et al., 2017; Koonin and Yutin, 2018). Nevertheless, different scenarios for the origin of giant viruses are still being debated (Forterre and Gaia, 2016).

Although the excitement over giant viruses has led to speculation and debate about their origins and evolutionary relationships with other viruses and cellular hosts, relatively few of these viruses have been isolated and characterized (Wilhelm et al., 2017). The collection of giant virus genomes also remains relatively small although expanded through recent efforts using amoeba and other protist hosts, complemented by metagenomics assembly (Wilhelm et al., 2016, 2017). To attain a definitive picture of giant virus evolution and ecology, additional efforts in both giant virus genomics and biology are essential. Concurrent with the progress in Mimivirus research, smaller viruses with core gene phylogenies that place them on the Mimivirus branch of the NCLDV have also been characterized (Santini et al., 2013; Yutin et al., 2013; Moniruzzaman et al., 2014). These smaller relatives of Mimiviruses have been called the “extended *Mimiviridae*” and infect single-celled eukaryotic algae but are phylogenetically distinct from the *Phycodnaviridae*, a virus family defined, in part, by their infection of algae (Van Etten and Meints, 1999; Dunigan et al., 2006; Wilson et al., 2009). As such, this algae-infecting, novel group of Mimivirus relatives represents a source of viral diversity that is both evolutionarily informative and environmentally relevant.

Recently, viruses infecting the freshwater algae *Chrysochromulina parva* were isolated from Lake Ontario (Mirza et al., 2015). Initial sequence analysis of the B-family DNA polymerase amplified from culture lysates indicated close phylogenetic relationship with the *Phycodnaviridae*, primarily Group II *Phaeocystis globosa* viruses (e.g., PgV-03T; Brussaard et al., 2004; Baudoux and Brussaard, 2005) and the prymnesiovirus CbV-PW1 which infects *Chrysochromulina brevifilum* (Suttle and Chan, 1995), making it the only other type of phycodnavirus, beside chloroviruses, to be described in freshwater ecosystems. However, sequencing of amplified major capsid protein genes from *C. parva* virus lysates revealed a diverse mix of genes from both the extended *Mimiviridae* and *Phycodnaviridae*, raising questions about the identity of *C. parva* viruses and even suggesting that more than one virus might infect this algal lineage (Mirza et al., 2015).

In this study, we isolated, sequenced, and characterized the genome of a *C. parva* virus that we named CpV-BQ2. During sequencing, we also captured and characterized the genomes of three putative virophages of the PLV group that we predict to exploit the infection cycle of CpVs to replicate. We used the genomic sequences obtained here to screen publicly available metatranscriptomic datasets from freshwater ecosystems in multiple locations and time points for the presence and activity of CpV-BQ2, observing substantial activity in both Lake Erie and Lake Tai, China (*Taihu* in Mandarin Chinese). Given that the virus was originally isolated from Lake Ontario (North America), this observation suggests that close relatives of CpV-BQ2 are globally distributed and are active players in freshwater ecosystems. This study establishes a foundation for future research with CpVs, which are useful models for studies of freshwater algal viruses and may foster a deeper understanding of the complex interactions of giant viruses with their algal hosts.

## Materials and Methods

### CpV Propagation and Purification

Viruses infecting the Prymnesiophyte algae *C. parva* CCMP 291 (a non-axenic strain) were originally isolated in 2011 (Mirza et al., 2015) and have been maintained in the laboratory since. To produce virus genomic material for sequencing, CpV lysates were generated from a series of 150 and 500 mL mid-log phase *C. parva* batch cultures grown at a constant temperature of 15°C, with a 12:12 h light-dark cycle at approximately 23 μE m^-2^ s^-1^ in DY-V medium (Andersen, 2005). The resulting lysates were filtered through 47-mm diameter, 0.50-μm nominal pore-size borosilicate glass microfiber Advantec^®^ filters (Life Science Products, Inc.) followed by filtration through 47-mm diameter, 0.22-μm pore-size PVDF Durapore^®^ membranes (EMD Millipore). The filtered lysates were concentrated approximately 200-fold *via* ultracentrifugation using a SW32Ti rotor (Beckman Coulter) as previously described (Short et al., 2011). After ultracentrifugation, the pelleted material was resuspended in 10 mM Tris–Cl (pH 8.5), pooled, and stored at 4°C. Filtered and concentrated lysates were further purified using Optiprep^TM^ (Iodixanol, Millipore Sigma Canada Co.) step gradients. Four-step gradients were created using Optiprep^TM^ solutions diluted in ultrapure H_2_O to final concentrations of 40, 35, 30, and 25% v/v, whereby 2.50 mL of each step was bottom loaded in 13.2 mL Ultra-Clear^TM^ ultracentrifuge tubes (Beckman Coulter Canada, LP) starting with the 25% solution and ending with the 40% solution following (Moniruzzaman et al., 2014). Three milliliters of concentrated lysate were then loaded on the top of the gradient which was ultracentrifuged in a SW40Ti rotor (Beckman Coulter) for 14.75 h at 39,000 rpm. Following ultracentrifugation, visible bands formed approximately one-third of the distance from the top of the tube, and 1.50 mL of this band and immediately surrounding gradient medium was collected by aspiration and was stored at 4°C.

### CpV DNA Extraction and Precipitation

Nucleic acids were extracted from gradient-purified bands using a QIAamp^®^ MinElute^®^ Virus Spin Kit (Qiagen) following the manufacturer’s recommendations with the following modifications: each MinElute column was loaded with lysed material twice, and 50 μL of Buffer AVE (RNase-free water with 0.04% sodium azide) was used during each elution step. To further concentrate purified genomic DNA, ethanol precipitation was conducted by mixing pooled, extracted DNA with 0.1× volume of 3 M NaOAc and 3× volume absolute ethanol followed by incubation at -20°C overnight. Precipitated DNA was then collected by centrifugation for 1 h at 14,000 × *g* at 4°C, the supernatant was decanted, and the DNA pellet was washed twice with ice-cold 70% ethanol. After being left to dry at room temperature, the DNA pellet was resuspended with pure H_2_O, and was stored at -20°C. DNA concentration was quantified using an Invitrogen^®^ Qubit^®^ 3.0 Fluorometer and dsDNA HS Assay kit (Thermo Fisher Scientific). In total 100 μL of DNA at a concentration of approximately 5 ng μL^-1^ was submitted to HudsonAlpha Institute for Biotechnology for sequencing.

### Quality Control, Sequence Assembly, and Annotation

Raw sequences were imported into the CLC Genomics Workbench v. 10.0.1 (Qiagen, Hilden, Germany) and processed for quality control. Reads below 0.03 quality score cutoff were removed from subsequent analyses, and the remaining reads were trimmed of any ambiguous and low quality 5′ bases and only reads at the full length were retained for assembly. Quality controlled reads were then assembled using the SPAdes 3.10.1 assembler with nine iterative kmer assemblies (kmers 21, 33, 55, 65, 77, 85, 99, 113, and 127) and the “careful option” turned on for contig correction. Scaffolds with length >5000 bp were then imported into CLC Genomics workbench for contig quality assessment and analysis. Quality controlled reads were mapped onto scaffolds with high stringency (>0.7 length fraction, >0.97 similarity fraction) to determine coverage. In order to reduce the number of scaffolds to only those of possible viral origin, scaffold libraries were aligned to the NCBI 16S rRNA gene database and hits were removed from future analyses. The remaining scaffolds were BLAST searched against a protein database downloaded from NCBI containing sequences from all currently sequenced giant virus genomes. Open reading frames were predicted using CLC Genomics workbench and coding sequences were imported into BLAST2GO for functional annotation. Open reading frames (ORFs) were plotted and whole genome alignments were generated using the BLAST Ring Image Generator (BRIG) (Alikhan et al., 2011). To spot check the CpV-BQ2 genome assembly, several PCR primer sets were designed and used to amplify DNA across tandem genes; i.e., the forward and reverse primers were designed so each would target a different gene. Further, these PCR confirmations were designed to focus on neighboring genes that were annotated as being derived from a different source (no hit, or closest hit being a gene from bacteria, eukaryotes, or other NCLDVs). During sequence assembly and annotation, contigs of putative virophages were also recovered and were translated with MetaGeneMark (Zhu et al., 2010). The resulting proteins were annotated using psi-blast searches (Altschul et al., 1997) with profiles generated from the alignments of the predicted proteins with homologous sequences from the conserved domain database and/or homologous proteins of PLV used as queries (Yutin et al., 2015).

### Phylogenetic Analysis

Reference amino acid sequences for virus B-family DNA polymerase (*polB*), A32-like virion packaging ATPase (ATPase), and the major capsid protein (MCP) were downloaded from the NCBI refseq database (see Supplementary Table 1 for *polB* accession numbers). These reference sequences were aligned with CpV-BQ2 coding sequence translations using MUSCLE (Edgar, 2004) in the MEGA v7.0.26 software package (Kumar et al., 2016). Following alignment, gapped columns (more than 30% of gaps) and columns with low information content were removed from the alignments as described previously (Yutin et al., 2008). The filtered alignments were used for tree reconstruction using PhyML3.0 (Guindon et al., 2010) with the LG substitution model, gamma-distributed site rates, empirical amino acid frequencies and aBayes branch support values. Sequences of the predicted capsid proteins of new virophages were combined with their closest relatives from the env_nr database (found by blastp), and those of previously identified PLVs (Yutin et al., 2015). Proteins were aligned using MUSCLE (Edgar, 2004), and gapped columns and columns with low information content were removed from the alignments before filtered alignments were used for tree reconstructions as described above for CpV sequences.

### Environmental Quantification and Statistical Analysis

To determine whether of CpV-BQ2 and its PLVs were active in freshwater systems, we used 4 publicly available metatranscriptome datasets from MG-RAST under project mgp82644^1^. These datasets were isolated from samples collected from Lake Erie and Lake Tai during cyanobacterial bloom seasons of 2013 and 2014 (Steffen et al., 2017; Stough et al., 2017). Quality filtered and trimmed reads were mapped to CpV-BQ2 (0.8 identity fraction, 0.7 length fraction) and the three PLV (0.8 identity fraction, 0.5 length fraction) genomes in CLC Genomics Workbench 10.0.1. Expression values were normalized per million reads within each library. Expression values were imported into the R statistical software package (R Core Team, 2015) and Spearman’s rank correlation coefficients were calculated using the hmisc package (Harrell, 2016). Data were visualized in SigmaPlot v.12.5 (Systat Software, Inc.).

## Results

### Assembly and Annotation of CpV-BQ2

**FIGURE 1:**
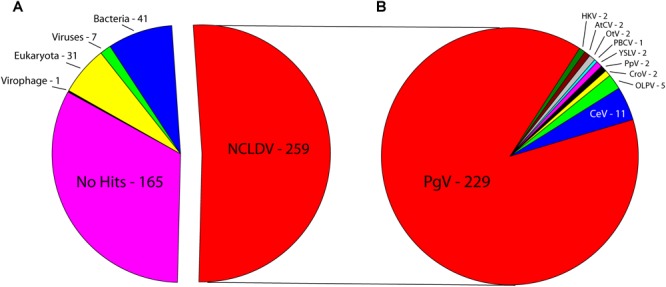
Best BLAST hits of CpV-BQ2 predicted open reading frames against **(A)** NCLDVs, other viruses, virophages, and the three domains of life. **(B)** Specific NCLDV representatives. PgV-14T – *Phaeocystis globosa* virus, CeV – *Chrysochromulina ericina* virus, OLPV – Organic Lake Phycodnavirus, CroV – *Cafeteria roenbergensis* virus, PpV – *Pyramimonas pouchetti* virus, YSLV – Yellowstone Lake Phycodnavirus, PBCV – *Paramecium bursaria Chlorella* virus, OtV – *Ostreococcus tauri* virus, AtCV – *Acanthamoeba turfacea Chlorella* virus, HKV – Hokovirus.

Sequencing on the Illumina MiSeq platform yielded 26,745,770 reads for genome assembly, which was reduced to 26,729,526 after quality control. The genome of *C. parva* virus BQ2 (accession MH918795) was stringently assembled using SPAdes with read correction and post-assembly scaffold checking. The result was 1099 scaffolds over 5000 bp in length, which were screened for the presence of NCLDV core genes. The largest fragment was a 437,255 bp scaffold with an average coverage of 127.44 and a GC content of 25%, encoding 503 predicted ORFs. Comparison of the sequences of the predicted CpV-BQ2 ORFs (Figure 1) showed that more than half had top BLAST hits to NCLDV genes, the vast majority of which were from group I *P. globosa* viruses (collectively termed PgV), but several showed the highest similarity to either phycodnavirus genes or to inferred genes of the metagenomics assembly of Hokovirus within the “Klosneuvirinae” subgroup of Mimiviruses (Schulz et al., 2017). The remaining genes with taxonomic assignments were split, primarily, between eukaryotes and bacteria, with a few showing the highest similarity to homologs from other viruses including virophages. At the time of our analysis, 165 of the ORFs had no significant BLAST hits. Without exception, the PCRs designed to confirm the genome assembly resulted in amplification of gene fragments corresponding to the expected sizes indicating that these gene fragments were physically linked on the same DNA molecule as predicted from the assembly (Supplementary Table 2).

**FIGURE 2:**
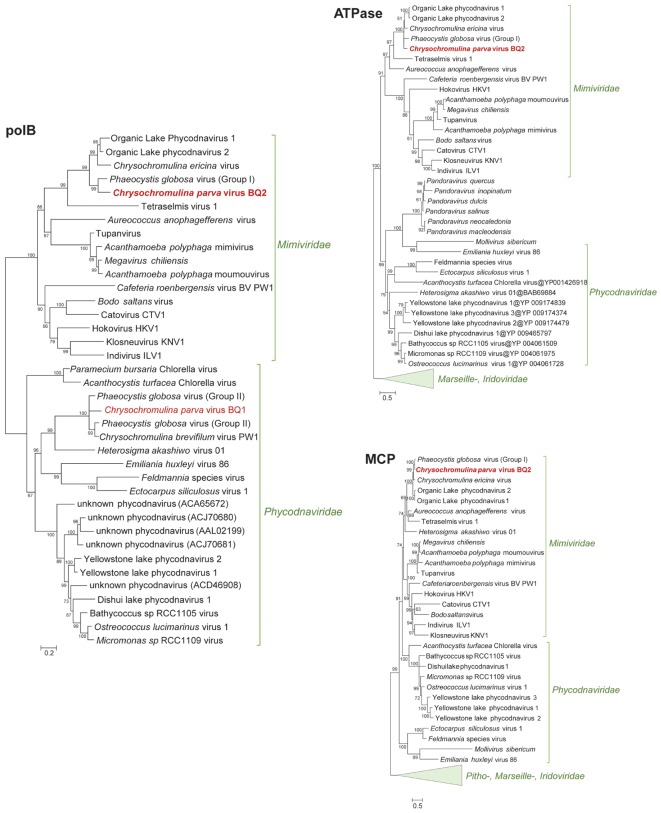
Approximately maximum-likelihood phylogenetic trees of B-family DNA polymerase (*polB*), A32-like virion packaging ATPase (ATPase), and the major capsid protein (MCP). The *polB* tree is constructed on protein fragments corresponding to PCR amplicons reported in Mirza et al. (2015). When more than one MCP gene was present in a genome (*Mimiviridae*), a paralog closest to *Phycodnaviridae* was chosen for the MCP tree. Node support (aLRT-SH statistic) >50% are shown. Accession numbers for the *polB* sequences are provided in Supplementary Table 1.

**FIGURE 3:**
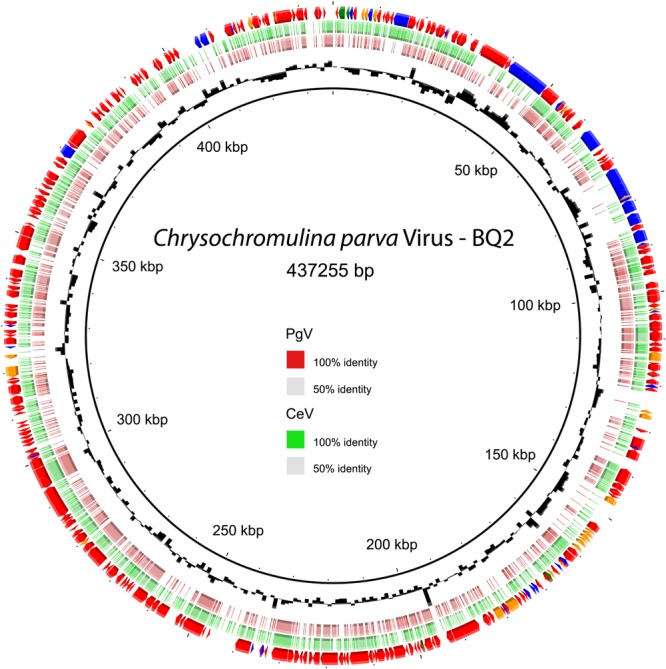
CpV-BQ2 ORF prediction and whole genome alignment of nucleotides. Outer ring – ORF prediction and top BLAST hit (Red arrows – NCLDV, Blue arrows – Bacteria, Orange arrows – Eukaryotes, Green arrows – Viruses). Second outermost ring in green – whole genome alignment with CeV where color gradient represents sites similar to CeV. Third outermost ring in red – whole genome alignment with PgV-14T where color gradient represents sites similar to PgV. Innermost ring in black – GC content. The genome is presented as a circle for the purpose of visualization and is not meant imply that the genome is a circular molecule. The 12 o’clock position represents the beginning/end of the genome.

Phylogenetic analysis of B-family DNA polymerase (*polB*), A32-like virion packaging ATPase (ATPase), and the major capsid protein (MCP) genes, all core NCLDV genes (Yutin et al., 2009), yielded similar results with PgV group I genes identified as the closest relatives to CpV-BQ2 (Figure 2). The remaining 7 of the 10 most common “core” NCLDV genes (Supplementary Figures 1–7) also showed similar phylogenies, with only two exceptions. The CpV-BQ2 RNA polymerase β-subunit was most closely related to Organic Lake Phycodnavirus 1, but with PgV as the next closest relative (Supplementary Figure 5), and the Superfamily II Helicase was closest to a poxvirus, but with extended *Mimiviridae* member *Aureococcus anophagefferens* virus (AaV) as the next closest relative (Supplementary Figure 2). Whole genome alignment of CpV with the two closest relatives, PgV and CeV, showed considerable similarity across the entire genome (Figure 3). Regions of low similarity and gaps shown in the alignment generally corresponded to predicted CpV ORFs that are not represented in the genomes of the related viruses and non-coding regions.

The functional annotation of the CpV-BQ2 genome (Figure 3) is limited, with no homologs with known or predicted function detected for 320 of the 503 ORFs. The annotated ORFs encode characteristic NCLDV proteins such as virion components (two paralogs of the Major Capsid Protein; CpV ORFs 105 and 177), as well as proteins involved in virion morphogenesis (A32-like packaging ATPase; CpV ORF 098), viral transcriptional regulation (late transcription factor VLTF3; CpV ORF 176), and DNA replication, repair, and nucleotide metabolism. The latter functional class included DNA polymerase, replication factor C, the primase-helicase fusion protein characteristic of the NCLDV, mismatch repair proteins (MutS7 and MutS8), and ribonucleotide reductase. Additionally, CpV-BQ2 encodes a large contingent of genes with predicted DNA modification activities compared to the related Mimiviruses, including 14 DNA methyltransferases and a histone acetyltransferase. The genome also features a group of five eukaryotic E3 ubiquitin ligases, three of which are clustered together in one location on the genome (CpV ORFs 167, 169, and 171). The annotated CpV-BQ2 genome sequence was submitted to GenBank (accession MH918795).

### Functional Potential and Phylogeny of the CpV-Associated Virophages (PLV)

**FIGURE 4:**
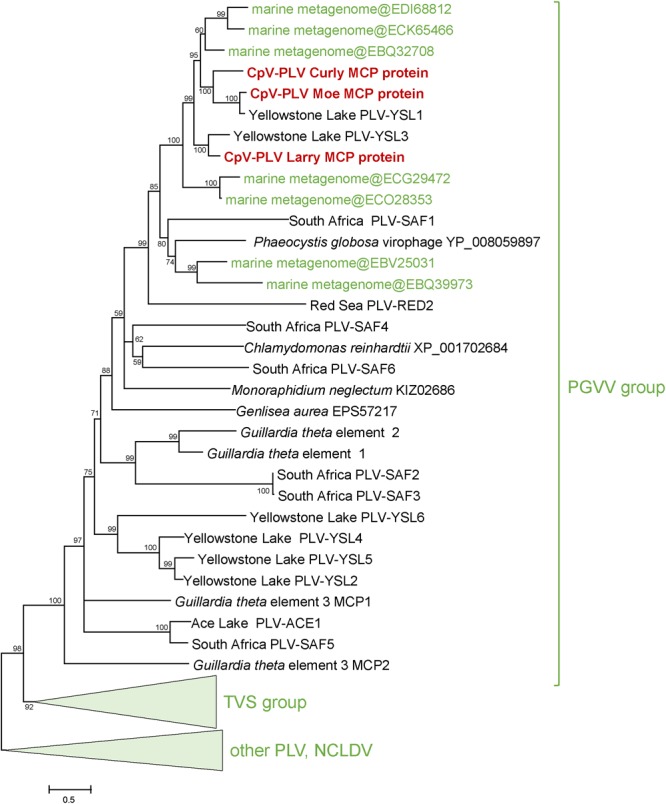
Phylogenetic tree for the CpV-PLV MCP genes. Sequences in green were retrieved from GenBank by blastp searches initiated from CpV-PLV MCP sequences. Other reference sequences are from Yutin et al. (2015). GenBank protein IDs are shown in parentheses (whenever available). The numbers at the internal branches indicate local likelihood-based support (percentage points). PgVV, *Phaeocystis globosa* virus virophage; SAF, South Africa; RED, Red Sea; MED, Mediterranean Sea; YSL, Yellowstone Lakes; TSV, *Tetraselmis viridis* virophage.

**FIGURE 5:**
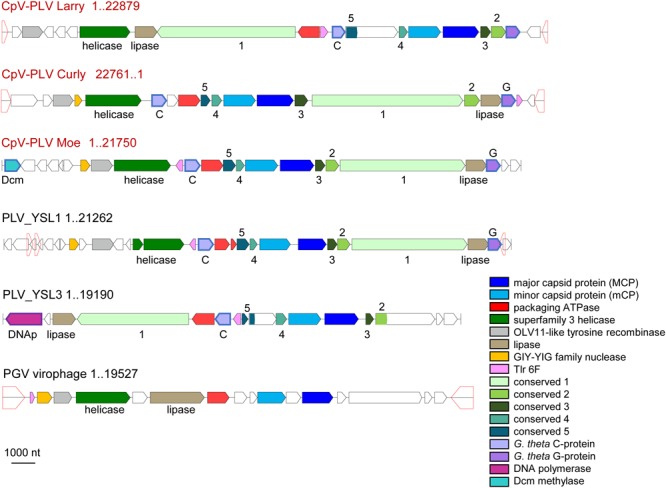
Genome architectures of the CpV-associated polintoviruses (CpV-PLVs). Homologous genes are color-coded, as shown in the inset. Homologous genes without predictable function (activity) are named as “conserved 1–5.” Open pentagons represent inverted repeats. CpV-PLV Curly shown as reverse complement. Reference virophages, as noted in Yutin et al. (2015), are abbreviated as: YSL1 and YSL3, Yellowstone Lake PLV 1 and 3; PgVV, *Phaeocystis globosa* virus virophage.

Three putative *C. parva* virus virophages of the PLV group were assembled along with the “host” virus genome. We designated these CpV-PLV Larry (MH920636), CpV-PLV Curly (accession MH919296), and CpV-PLV Moe (MH919297). CpV-PLV Curly is 22,761 bases long with a GC content of 37.8% and ∼42,000× average coverage. The coverage is the highest in the assembly, about sixfold higher than the next highest contig, and 1000-fold higher than the majority of contigs. Phylogenetic analysis of the major capsid protein (MCP) places all three CpV-PLVs within the *P. globosa* virus virophage (PgVV) group of the PLVs (Figure 4). In accord with this phylogenetic position, CpV-PLV have gene repertoires and genome architectures characteristic of the PGVV group (Figure 5; Yutin et al., 2015). Besides the hallmark PLV genes (major and minor capsid proteins, packaging ATPase, superfamily three helicase, OLV11-like tyrosine recombinase), CPV-PLVs encode five uncharacterized conserved proteins that are shared only with the Yellowstone Lake PLVs that were assembled by culture independent methods (PLV_YSL1 and PLV_YSL3)^2^.

CpV-PLV Curly encodes 19 ORFs (Figure 4), of which 8 have predicted functions and 7 have a conserved domain of unknown function. The genome also encodes a minor capsid protein (CpV-PLV ORF 11), and a major capsid (CpV-PLV ORF 12) which appears to be related to the Yellowstone Lake virophage meta-assemblies 1 and 3, grouped with a number of uncultured PLVs assembled from metagenomes (Figure 5). CpV-PLV Curly ORF 17 encodes a hypothetical protein with similarity to the Qinghai Lake Virophage meta-assembly gene QLV_03, and CpV-PLV Curly ORF 18 shows similarity to mobile elements present in *Guillardia theta, Muricauda* sp., and *Tetrahymena thermophila* genomes. Apart from the expected virophage genes, CpV-PLV Curly also encodes a predicted HNH homing endonuclease and DNA-methyltransferase, the top nr-BLAST search hits for both of which are bacterial. One additional open reading frame encodes a predicted E3 ubiquitin ligase. CpV-PLV Moe is closely similar to Curly, with a 21,750 bp genome, 30.1% GC, and 23 predicted ORFs. Unlike the other two PLVs, CpV-PLV Moe also appears to encode a putative DNA cytosine methyltransferase. CpV-PLV Larry is distinct from the other two, with a 22,879 bp genome, 39.3% GC, and 20 predicted ORFs, possessing the same core elements as the other two, but with the 5′ half of the genome inverted. It should also be noted that most of the predicted ORFs exhibit similar order to the orthologs in YSL1 and PgVV (Figure 4). GenBank accession numbers for the annotated virophage genomes for CpV-PLV Larry, Curly and Moe are MH920636, MH919296, and MH919297, respectively.

### Environmental Abundance of CpV and CpV PLV

**FIGURE 6:**
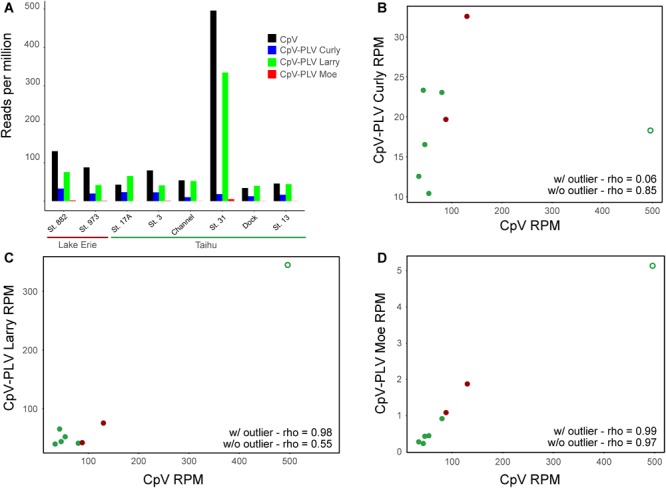
**(A)** Metatranscriptomic sequencing reads sequenced from the Lake Erie and Lake Tai from 2013 cyanobacterial blooms mapped to CpV-BQ2 and virophage genomes and expressed in reads per million. Spearman’s correlation coefficients (rho) are shown for data including the outlier sample and for data excluding outlier sample for the correlations between reads mapped to CpV-BQ2 and **(B)** CpV-PLV Curly, **(C)** CpV-PLV Larry, and **(D)** CpV-PLV Moe. Points in panels **(B–D)** are colored red or green to show data points collected from Lake Erie or Tai, respectively, and the outlier data points are denoted with open circles.

To determine whether genes of CpV-BQ2 and CpV-PLVs are expressed in lake systems, currently available environmental metatranscriptome reads from environmental datasets were mapped to the CpV-BQ2 and CpV-PLV genomes with a minimum length cutoff of 0.8 and similarity fractions of 0.8 and 0.5, respectively. Overall, 4 metatranscriptomes isolated from freshwater ecosystems during *Microcystis aeruginosa* blooms were initially mapped. *Microcystis* is a cyanobacterial genus responsible for causing severe, environmentally disruptive blooms globally and is often capable of producing the potent hepatotoxin microcystin. Samples were collected from the hypereutrophic Lake Tai (*Taihu* in Mandarin) in China during the bloom seasons of 2013 and 2014, while the remaining 2 metatranscriptomic groups were sampled from blooms in Lake Erie, United States during the same years. Of these data sets, metatranscriptomes sequenced from Lake Erie and Lake Tai during 2013 showed a moderate level of recruitment with good coverage of CpV-BQ2, CpV-PLV Curly, and CpV-PLV Larry genomes across all samples with a spike in activity in the *Taihu* Station 31 sample (Figure 6A), however, transcript abundance was too low to discern distinct transcriptional patterns from more than this sample. Reads mapped to the genome of CpV-BQ2 were highly correlated with CpV-PLV Larry (Figure 6C; rho = 0.98, *p* = 9.768 × 10^-9^) and Moe (Figure 6D; rho = 0.99, *p* = 1.8 × 10^-9^), but not Curly (Figure 6B; rho = 0.06, *p* = 0.823). In order to verify that the sample obtained from Taihu Station 31 was not driving the observed correlations on its own, we removed the sample and re-tested correlations. CpV-PLVs Larry and Moe read recruitment were both still significantly correlated to that of CpV-BQ2 (Larry: rho = 0.55, *p* = 0.026; Moe: rho = 0.99, *p* = 5.19 × 10^-6^), and correlation by CpV-PLV Curly increased when the outlier was removed from the analysis (rho = 0.65, *p* = 0.022).

## Discussion

In this study, we report the genome sequence and functional potential of a *C. parva* virus and its associated virophages that belong to the PLV group. CpVs were initially isolated and cultured from Lake Ontario, and amplification and sequencing yielded a confounding mixture of major capsid protein genes from different giant virus lineages (*Phycodnaviridae* and extended *Mimiviridae*), yet only a single phycodnavirus *polB* sequence (Mirza et al., 2015). To resolve this conundrum, whole genome sequencing was required. Surprisingly, based on differences in the *polB* sequence encoded in the assembled genome reported here, it is apparent that the genome sequence was recovered from a virus that is distinct from the originally described virus called CpV-BQ1 (Mirza et al., 2015). Because the CpV genome described here was obtained from the same samples as CpV-BQ1 but encodes a distinct *polB* gene, we named the *C. parva*-infecting algal mimi-like virus (member of the extended *Mimiviridae*) CpV-BQ2. To obtain a first glimpse into the ecology of CpV-BQ2 and its virophages, we used the assembled CpV-BQ2 and CpV-PLV genomes and extant environmental metatranscriptomic data to examine the activity of this virus and virophage consortium in two unique freshwater environments, Lake Erie and Lake Tai.

The size of the assembled CpV-BQ2 genome (∼437 kb) is at least 90% of the length of the genome size estimated from pulse-field gel electrophoresis (∼485 kb) as reported in Mirza et al. (2015), and whole genome alignments with CeV and PgV also suggest the genome is nearly complete (although this is difficult to validate). The whole genome alignment with PgV also suggests the genome is linear, though further experimentation will be required to resolve the ends of the genome and determine if the CpV genome is in fact linear. The genome content suggests that CpV-BQ2 is a versatile giant virus with a close evolutionary relationship to the marine algal members of the extended *Mimiviridae*, namely, PgV14T (group I) and CeV, as opposed to group II PgV viruses that belong to the *Phycodnaviridae*. This relationship is demonstrated by the phylogenies of the core genes and is buttressed by genomic comparison. Indeed, CpV-BQ2 shares a similar genome size, GC content, and content with group I PgVs and CeV, and for more than half of the CpV-BQ2 ORFs, the most similar homologs are found in these viruses. Like other members of the extended *Mimiviridae*, CpV-BQ2 also possesses a mosaic assortment of genes with closest homologs in other viruses, bacteria and eukaryotes. Moreover, as in other giant viruses, a large fraction of CpV-BQ2 genes have no readily detectable homologs.

In addition to the CpV-BQ2 genome, we sequenced the genomes of three putative virophages (Yutin et al., 2015). Each of these closely related virophages are PLV in the PgVV group, which suggests that PLV-type virophages are a characteristic of the extended *Mimiviridae* group of NCLDV and, at least, to some extent, coevolve with their large virus hosts. Given that no virophage particles were observed during the initial isolation of CpVs (Mirza et al., 2015) it is possible the virophage particles or genomes could be packaged within the CpV-BQ2 virion as suggested for PgVV (Santini et al., 2013). Presently, there is no information about the nature of the CpV-PLVs besides the fact that their genomic material was co-purified along with DNA from CpV-BQ2, and they encode putative major and minor capsid proteins. Hence, it is premature to speculate if these PLVs exist as pro-virophages or if they are encapsidated within CpV-BQ2 virions.

The gene repertoire of CpV-BQ2 includes several distinct features such as set of 23 genes involved in DNA and chromatin modification, including 13 predicted DNA methyltransferases, a histone demethylase, a histone acetyl transferase, and at least 8 predicted restriction-modification systems. In addition, the CpV-PLVs also encode a DNA methyltransferase. Methyltransferases have been implicated in the biology of giant viruses previously, given that genomes of some *Chlorella* viruses are heavily methylated and encode many methyltransferases that, in two cases, are accompanied by restriction endonucleases (Nelson et al., 1998; Etten and Meints, 1999). DNA methyltransferases have been identified in the genomes of other giant viruses as well although generally not accompanied by endonucleases. The functions of restriction-modification systems and solo methyltranferases in NCLDV are unknown, but involvement in counter-defense as well as inter-virus competition appears likely. Of similar interest is the presence of putative histone modification enzymes, histone demethylase (CpV-BQ2 ORF 120) and histone acetyltransferase (CpV-BQ2 ORF 503). Because neither CpV-BQ2 nor other members of extended *Mimiviridae* appear to encode histones, the role of the histone modification enzymes might include remodeling of the host chromatin and regulation of the host gene transcription (Kouzarides, 2007).

In addition to the expanded DNA modification machinery, an unusual feature of CpV-BQ2 is a cluster of five E3 ubiquitin ligases, all with closest homologs in eukaryotes, three of which are grouped within 1 kb of one another. Similar RING-finger E3 ubiquitin ligases have been observed in several Mimiviruses (Iyer et al., 2006), but they are generally fewer in number and are spread across the genome. Only *A. anophagefferens* virus (AaV) encodes a similar group of E3 proteins (Moniruzzaman et al., 2014). Although the functions of the ubiquitin ligases in the NCLDVs remain to be studied, they have been hypothesized to inhibit host cell defenses (Iyer et al., 2006; Chaurushiya et al., 2012). CpV-PLV Curly also encodes an E3 ubiquitin ligase which so far has not been detected in other virophages. Conceivably, this ubiquitin ligase could modify either virus or cellular host proteins, perhaps protecting the giant virus from the host defenses.

To explore the potential ecological role of CpV-BQ2 and its virophages in freshwater ecosystems, we mapped currently available metatranscriptome reads isolated and sequenced from *M. aeruginosa* blooms in Lake Erie and Lake Tai, China during the years 2013 and 2014. Expression was detected in both Lake Erie and Lake Tai during 2013 across all samples. Reads mapping to all three CpV-PLVs co-occurred with CpV-BQ2 in all samples, exhibiting a high correlation to those of the “host” virus. Considering that the metatranscriptomes used here were isolated from microbial communities dominated by freshwater cyanobacteria, and were not poly-A selected, activity by CpV-BQ2 must have been present at considerable abundances to be detected. Moreover, as infection cycles in environmental datasets are not synchronized, the relationship between CpV-BQ2 and its virophages must be close to be observed. Indeed, some of this complexity is reflected in sample Taihu Station 31 in which expression of both virus and virophage genes was considerably higher and correlative relationships shifted. As CpVs were originated from Lake Ontario, Canada (Mirza et al., 2015), it is likely that the virus and virophage observed in Lake Tai are not identical to our isolates but instead relatives. Despite potential differences in physiology, however, these results suggest that closely related freshwater algal mimiviridae-like viruses are globally distributed and environmentally relevant.

The results presented here suggest that CpV-BQ2 is an abundant and active member of the extended-*Mimiviridae* with a unique functional potential. As the first freshwater representative of the extended-*Mimiviridae* to be isolated and maintained in culture, CpV-BQ2 stands as an important model virus for the future. The CpV-associated virophages, although related to PgVV, present only the second cases of cultured virophages from the PLV group of viruses and offer an opportunity to study the giant virus-infecting-viruses in culture.

## Author Contributions

JS, SW, and SS designed the research project and wrote the manuscript. JB, JS, and YC performed the experiments. EG, EK, HP, JS, MM, MS, and NY analyzed the data. All authors participated in manuscript review and editing.

## Conflict of Interest Statement

The authors declare that the research was conducted in the absence of any commercial or financial relationships that could be construed as a potential conflict of interest.

## Footnotes

1 https://www.mg-rast.org/linkin.cgi?project=mgp82644

2 ftp://ftp.ncbi.nih.gov/pub/yutinn/CpV_2018/ for multiple protein alignments

## References

[B1] Alikhan N. F. Petty N. K. Ben Zakour N. L. Beatson S. A. (2011). BLAST Ring Image Generator (BRIG): simple prokaryote genome comparisons. *BMC Genomics* 12:10. 10.1186/1471-2164-12-402 21824423 PMC3163573

[B2] Altschul S. F. Madden T. L. Schaffer A. A. Zhang J. Zhang Z. Miller W. (1997). Gapped BLAST and PSI-BLAST: a new generation of protein database search programs. *Nucleic Acids Res.* 25 3389–3402. 10.1093/nar/25.17.3389 9254694 PMC146917

[B3] Andersen R. A. (2005). *Algal Culturing Techniques.* Amsterdam: Elsevier.

[B4] Baudoux A. C. Brussaard C. P. D. (2005). Characterization of different viruses infecting the marine harmful algal bloom species *Phaeocystis globosa*. *Virology* 341 80–90. 10.1016/j.virol.2005.07.002 16081120

[B5] Brussaard C. P. D. Short S. M. Frederickson C. M. Suttle C. A. (2004). Isolation and phylogenetic analysis of novel viruses infecting the phytoplankton *Phaeocystis globosa* (Prymnesiophyceae). *Appl. Environ. Microbiol.* 70 3700–3705. 10.1128/AEM.70.6.3700-3705.2004 15184176 PMC427783

[B6] Brussaard C. P. D. Wilhelm S. W. Thingstad F. Weinbauer M. G. Bratbak G. Heldal M. (2008). Global-scale processes with a nanoscale drive: the role of marine viruses. *ISME J.* 2 575–578. 10.1038/ismej.2008.31 18385772

[B7] Chaurushiya M. S. Lilley C. E. Aslanian A. Meisenhelder J. Scott D. C. Landry S. (2012). Viral E3 ubiquitin ligase-mediated degradation of a cellular E3: viral mimicry of a cellular phosphorylation mark targets the RNF8 FHA domain. *Mol. Cell* 46 79–90. 10.1016/j.molcel.2012.02.004 22405594 PMC3648639

[B8] Claverie J. M. Abergel C. (2010). Mimivirus: the emerging paradox of quasi-autonomous viruses. *Trends Genet.* 26 431–437. 10.1016/j.tig.2010.07.003 20696492

[B9] Colson P. de Lamballerie X. Fournous G. Raoult D. (2012). Reclassification of giant viruses composing a fourth domain of life in the new order megavirales. *Intervirology* 55 321–332. 10.1159/000336562 22508375

[B10] Colson P. de Lamballerie X. Yutin N. Asgari S. Bigot Y. Bideshi D. K. (2013). “Megavirales”, a proposed new order for eukaryotic nucleocytoplasmic large DNA viruses. *Arch. Virol.* 158 2517–2521. 10.1007/s00705-013-1768-6 23812617 PMC4066373

[B11] Dunigan D. D. Fitzgerald L. A. Van Etten J. L. (2006). Phycodnaviruses: a peek at genetic diversity. *Virus Res.* 117 119–132. 10.1016/j.virusres.2006.01.024 16516998

[B12] Edgar R. C. (2004). MUSCLE: a multiple sequence alignment method with reduced time and space complexity. *BMC Bioinformatics* 5:113. 10.1186/1471-2105-5-113 15318951 PMC517706

[B13] Etten J. L. V. Meints R. H. (1999). Giant viruses infecting algae. *Annu. Rev. Microbiol.* 53 447–494. 10.1146/annurev.micro.53.1.447 10547698

[B14] Filee J. Pouget N. Chandler M. (2008). Phylogenetic evidence for extensive lateral acquisition of cellular genes by nucleocytoplasmic large DNA viruses. *BMC Evol. Biol.* 8:13. 10.1186/1471-2148-8-320 19036122 PMC2607284

[B15] Fischer M. G. Suttle C. A. (2011). A virophage at the origin of large DNA transposons. *Science* 332 231–234. 10.1126/science.1199412 21385722

[B16] Forterre P. Gaia M. (2016). Giant viruses and the origin of modern eukaryotes. *Curr. Opin. Microbiol.* 31 44–49. 10.1016/j.mib.2016.02.001 26894379

[B17] Gaia M. Benamar S. Boughalmi M. Pagnier I. Croce O. Colson P. (2014). Zamilon, a novel virophage with mimiviridae host specificity. *PLoS One* 9:8. 10.1371/journal.pone.0094923 24747414 PMC3991649

[B18] Guindon S. Dufayard J. F. Lefort V. Anisimova M. Hordijk W. Gascuel O. (2010). New algorithms and methods to estimate maximum-likelihood phylogenies: assessing the performance of PhyML 3.0. *Syst. Biol.* 59 307–321. 10.1093/sysbio/syq010 20525638

[B19] Harrell F. E. Jr. (2016). *Hmisc: Harrell Miscellaneous. R Package Version 3.17–4*.

[B20] Iyer L. M. Aravind L. Koonin E. V. (2001). Common origin of four diverse families of large eukaryotic DNA viruses. *J. Virol.* 75 11720–11734. 10.1128/JVI.75.23.11720-11734.2001 11689653 PMC114758

[B21] Iyer L. M. Balaji S. Koonin E. V. Aravind L. (2006). Evolutionary genomics of nucleo-cytoplasmic large DNA viruses. *Virus Res.* 117 156–184. 10.1016/j.virusres.2006.01.009 16494962

[B22] Koonin E. Yutin N. (2018). Multiple evolutionary origins of giant viruses. *F1000 Res.* 7:1840. 10.12688/f1000research.16248.1 30542614 PMC6259494

[B23] Koonin E. V. Yutin N. (2010). Origin and evolution of eukaryotic large nucleo-cytoplasmic DNA viruses. *Intervirology* 53 284–292. 10.1159/000312913 20551680 PMC2895762

[B24] Kouzarides T. (2007). Chromatin modifications and their function. *Cell* 128 693–705. 10.1016/j.cell.2007.02.005 17320507

[B25] Krupovic M. Koonin E. V. (2015). Polintons: a hotbed of eukaryotic virus, transposon and plasmid evolution. *Nat. Rev. Microbiol.* 13:105. 10.1038/nrmicro3389 25534808 PMC5898198

[B26] Kumar S. Stecher G. Tamura K. (2016). MEGA7: molecular evolutionary genetics analysis version 7.0 for bigger datasets. *Mol. Biol. Evol.* 33 1870–1874. 10.1093/molbev/msw054 27004904 PMC8210823

[B27] La Scola B. Desnues C. Pagnier I. Robert C. Barrassi L. Fournous G. (2008). The virophage as a unique parasite of the giant mimivirus. *Nature* 455 100–U165. 10.1038/nature07218 18690211

[B28] Mirza S. F. Staniewski M. A. Short C. M. Long A. M. Chaban Y. V. Short S. M. (2015). Isolation and characterization of a virus infecting the freshwater algae *Chrysochromulina parva*. *Virology* 486 105–115. 10.1016/j.virol.2015.09.005 26432023

[B29] Moniruzzaman M. LeCleir G. R. Brown C. M. Gobler C. J. Bidle K. D. Wilson W. H. (2014). Genome of brown tide virus (AaV), the little giant of the Megaviridae, elucidates NCLDV genome expansion and host–virus coevolution. *Virology* 466(Suppl. C) 60–70. 10.1016/j.virol.2014.06.031 25035289

[B30] Moreira D. Brochier-Armanet C. (2008). Giant viruses, giant chimeras: the multiple evolutionary histories of Mimivirus genes. *BMC Evol. Biol.* 8:12. 10.1186/1471-2148-8-12 18205905 PMC2263039

[B31] Moreira D. Lopez-Garcia P. (2005). Comment on “The 1.2-megabase genome sequence of Mimivirus”. *Science* 308:3. 10.1126/science.1110820 15905382

[B32] Moreira D. Lopez-Garcia P. (2009). Ten reasons to exclude viruses from the tree of life. *Nat. Rev. Microbiol.* 7 306–311. 10.1038/nrmicro2108 19270719

[B33] Nelson M. Burbank D. E. Van Etten J. L. (1998). Chlorella viruses encode multiple DNA methyltransferases. *Biol. Chem.* 379 423–428. 10.1515/bchm.1998.379.4-5.423 9628333

[B34] R Core Team (2015). *R: A Language and Environment for Statistical Computing.* Vienna: R Foundation for Statistical Computing.

[B35] Raoult D. Audic S. Robert C. Abergel C. Renesto P. Ogata H. (2004). The 1.2-megabase genome sequence of mimivirus. *Science* 306 1344–1350. 10.1126/science.1101485 15486256

[B36] Santini S. Jeudy S. Bartoli J. Poirot O. Lescot M. Abergel C. (2013). Genome of Phaeocystis globosa virus PgV-16T highlights the common ancestry of the largest known DNA viruses infecting eukaryotes. *Proc. Natl. Acad. Sci. U.S.A.* 110 10800–10805. 10.1073/pnas.1303251110 23754393 PMC3696832

[B37] Schulz F. Yutin N. Ivanova N. N. Ortega D. R. Lee T. K. Vierheilig J. (2017). Giant viruses with an expanded complement of translation system components. *Science* 356 82–85. 10.1126/science.aal4657 28386012

[B38] Schvarcz C. R. Steward G. F. (2018). A giant virus infecting green algae encodes key fermentation genes. *Virology* 518 423–433. 10.1016/j.virol.2018.03.010 29649682

[B39] Short S. M. Rusanova O. Staniewski M. A. (2011). Novel phycodnavirus genes amplified from Canadian freshwater environments. *Aquat. Microb. Ecol.* 63 61–67. 10.3354/ame01478

[B40] Steffen M. M. Davis T. W. McKay R. M. Bullerjahn G. S. Krausfeldt L. E. Stough J. M. A. (2017). Ecophysiological examination of the Lake Erie Microcystis bloom in 2014: linkages between biology and the water supply shutdown of Toledo, Ohio. *Environ. Sci. Technol.* 51 6745–6755. 10.1021/acs.est.7b00856 28535339

[B41] Stough J. M. A. Tang X. M. Krausfeldt L. E. Steffen M. M. Gao G. Boyer G. L. (2017). Molecular prediction of lytic *vs* lysogenic states for *Microcystis* phage: metatranscriptomic evidence of lysogeny during large bloom events. *PLoS One* 12:17. 10.1371/journal.pone.0184146 28873456 PMC5584929

[B42] Suttle C. A. Chan A. M. (1995). Viruses infecting the marine prymnesiophyte *Chrysochromulina* spp.: isolation, preliminary characterization and natural abundance. *Mar. Ecol. Prog. Ser.* 118 275–282. 10.3354/meps118275

[B43] Van Etten J. L. Meints R. H. (1999). Giant viruses infecting algae. *Annu. Rev. Microbiol.* 53 447–494. 10.1146/annurev.micro.53.1.447 10547698

[B44] Wilhelm S. Bird J. Bonifer K. Calfee B. Chen T. Coy S. (2017). A student’s guide to giant viruses infecting small eukaryotes: from acanthamoeba to zooxanthellae. *Viruses* 9:46. 10.3390/v9030046 28304329 PMC5371801

[B45] Wilhelm S. W. Coy S. R. Gann E. R. Moniruzzaman M. Stough J. M. A. (2016). Standing on the shoulders of giant viruses: five lessons learned about large viruses infecting small eukaryotes and the opportunities they create. *PLoS Pathog.* 12:5. 10.1371/journal.ppat.1005752 27559742 PMC4999288

[B46] Wilson W. H. Van Etten J. L. Allen M. J. (2009). The *phycodnaviridae*: the story of how tiny giants rule the world. *Curr. Top. Microbiol. Immunol.* 328 1–42. 10.1007/978-3-540-68618-7_1 19216434 PMC2908299

[B47] Yutin N. Colson P. Raoult D. Koonin E. V. (2013). Mimiviridae: clusters of orthologous genes, reconstruction of gene repertoire evolution and proposed expansion of the giant virus family. *Virol. J.* 10:106. 10.1186/1743-422X-10-106 23557328 PMC3620924

[B48] Yutin N. Makarova K. S. Mekhedov S. L. Wolf Y. I. Koonin E. V. (2008). The deep archaeal roots of eukaryotes. *Mol. Biol. Evol.* 25 1619–1630. 10.1093/molbev/msn108 18463089 PMC2464739

[B49] Yutin N. Shevchenko S. Kapitonov V. Krupovic M. Koonin E. V. (2015). A novel group of diverse Polinton-like viruses discovered by metagenome analysis. *BMC Biol.* 13:95. 10.1186/s12915-015-0207-4 26560305 PMC4642659

[B50] Yutin N. Wolf Y. I. Koonin E. V. (2014). Origin of giant viruses from smaller DNA viruses not from a fourth domain of cellular life. *Virology* 466 38–52. 10.1016/j.virol.2014.06.032 25042053 PMC4325995

[B51] Yutin N. Wolf Y. I. Raoult D. Koonin E. V. (2009). Eukaryotic large nucleo-cytoplasmic DNA viruses: clusters of orthologous genes and reconstruction of viral genome evolution. *Virol. J.* 6:223. 10.1186/1743-422X-6-223 20017929 PMC2806869

[B52] Zhu W. Lomsadze A. Borodovsky M. (2010). Ab initio gene identification in metagenomic sequences. *Nucleic Acids Res.* 38:e132. 10.1093/nar/gkq275 20403810 PMC2896542

