# Corrigendum: Genome and Environmental Activity of a *Chrysochromulina parva* Virus and Its Virophages

**DOI:** 10.3389/fmicb.2019.00907

**Published:** 2019-04-24

**Authors:** Joshua M. A. Stough, Natalya Yutin, Yuri V. Chaban, Mohammed Moniruzzaman, Eric R. Gann, Helena L. Pound, Morgan M. Steffen, Jenna N. Black, Eugene V. Koonin, Steven W. Wilhelm, Steven M. Short

**Affiliations:** ^1^Department of Microbiology, The University of Tennessee, Knoxville, Knoxville, TN, United States; ^2^National Center for Biotechnology Information, National Library of Medicine, National Institutes of Health, Bethesda, MD, United States; ^3^Department of Biology, University of Toronto Mississauga, Mississauga, ON, Canada; ^4^Department of Biology, James Madison University, Harrisonburg, VA, United States

**Keywords:** giant viruses, algae, NCLDV, freshwater, virophage, genome

In the original article, there was a mistake in Figure 2 as published. In the figure, the tree branch closest to *Chrysochromulina parva* virus BQ2 labeled as “*Phaeocystis globosa* virus (group II)” should have been labeled “*Phaeocystis globosa* virus (group I).” Similarly, the two branches labeled as “*Phaeocystis globosa* virus (group I)” and “*Phaeocystis globosa* virus” adjacent to *Chrysochromulina parva* virus BQ1 should have both been labeled as “*Phaeocystis globosa* virus (group II).”

**Figure 2:**
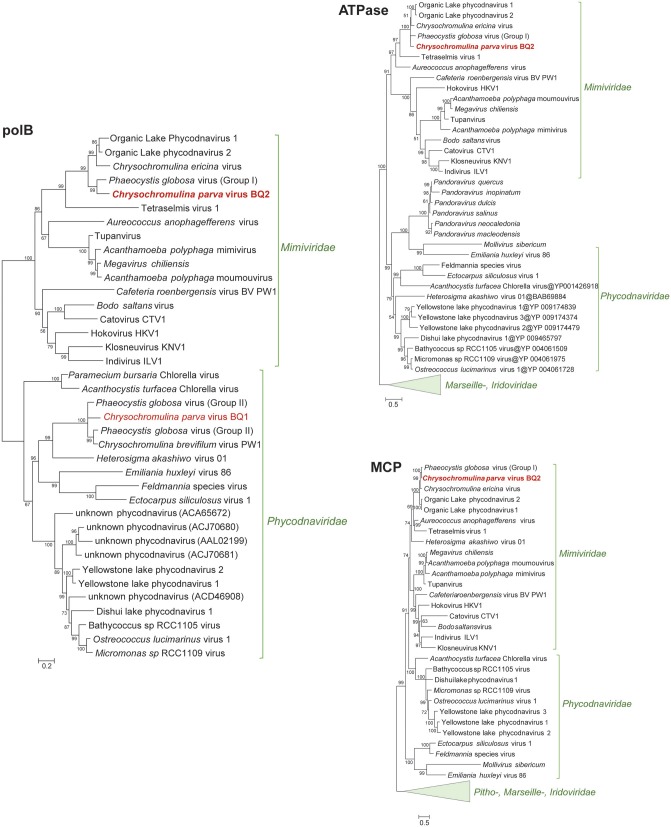
Approximately maximum-likelihood phylogenetic trees of B-family DNA polymerase (*polB*), A32-like virion packaging ATPase (ATPase), and the major capsid protein (MCP). The *polB* tree is constructed on protein fragments corresponding to PCR amplicons reported in Mirza et al. (2015). When more than one MCP gene was present in a genome (*Mimiviridae*), a paralog closest to *Phycodnaviridae* was chosen for the MCP tree. Node support (aLRT-SH statistic) >50% are shown. Accession numbers for the *polB* sequences are provided in Supplementary Table 1.

The authors apologize for this error and state that this does not change the scientific conclusions of the article in any way. The original article has been updated.
