# Supplementary material for: Genome and Environmental Activity of a Chrysochromulina parva Virus and Its Virophages
Source: Front Microbiol. 2019 Apr 5;10:703. doi: 10.3389/fmicb.2019.00703 (PMC6459981; doi:10.3389/fmicb.2019.00703)
Supplement: Supplementary file 1 [file Data_Sheet_1.PDF]

Supplementary Material

Genome and environmental activity of a *Chrysochromulina parva* virus and its virophages

Joshua M.A. Stough[1], Natalya Yutin[2], Yuri V. Chaban[3], Mohammed Moniruzzaman[1], Eric R. Gann[1], Helena L. Pound[1], Morgan M. Steffen[4], Jenna N. Black[3], Eugene V. Koonin[2], Steven W. Wilhelm[1*], Steven M. Short[3*]

***correspondence:** Steven M. Short: steven.short@utronto.ca, Steven W. Wilhelm: wilhelm@utk.edu.

Supplementary Table 1. GenBank accession numbers for reference sequences used in the polB phylogenetic tree.

Reference Sequence Name	GenBank Accession Number
Micromonas sp RCC1109 virus	YP_004062103
Ostreococcus lucimarinus virus 1	YP_004061851
Bathycoccus sp RCC1105 virus	YP_004061614
unknown phycodnavirus	ACD46908
Yellowstone lake phycodnavirus 1	YP_009174732
Yellowstone lake phycodnavirus 2	YP_009174598
unknown phycodnaviruses	AAL02199, ACA65672, ACJ70680, ACJ70681
Dishui lake phycodnavirus 1	YP_009465906
Ectocarpus siliculosus virus 1	NP_077578
Feldmannia species virus	YP_002154715
Emiliania huxleyi virus 86	YP_293784
Heterosigma akashiwo virus 01	BAE06251
Chrysochromulina brevifilum virus PW1	AAB49739
Phaeocystis globosa virus (Group II)	ABD62757
Chrysochromulina parva virus BQ1	ALH45652
Phaeocystis globosa virus (Group II)	AAR05089
Acanthocystis turfacea Chlorella virus	YP_001427279
Paramecium bursaria Chlorella virus	NP_048532
Organic Lake phycodnavirus 1	ADX06143
Organic Lake phycodnavirus 2	ADX06483
Chrysochromulina ericina virus	A7U6F1
Chrysochromulina parva virus BQ2	This study (ORF_301)
Phaeocystis globosa virus (Group I)	YP_008052566
Tetraselmis virus 1	AUF82649
Cafeteria roenbergensis virus BV PW1	YP_003970183
Indivirus ILV1	ARF09771
Klosneuvirus KNV1	ARF11832, ARF11831
Hokovirus HKV1	ARF11096
Catovirus CTV1	ARF09278
Bodo saltans virus	MF782455
Aureococcus anophagefferens virus	YP_009052217
Tupanvirus	AUL77893
Acanthamoeba polyphaga mimivirus	YP_003986825
Acanthamoeba polyphaga moumouvirus	AEX62677
Megavirus chiliensis	YP_004894633

Supplementary Table 2. PCR amplifications conducted across multiple ORFs to spot-check CpV-BQ2 genome assembly.

**ORFs Targeted**	**Gene Types***	**Amplicon Positions & Expected Size**	**Length of Amplified Fragment**
ORF 300 (hypothetical protein) – ORF 301 (DNA polymerase)	unknown – NCLDV	273127-274605 (1478 bp)	~1450 bp
ORF 301 (DNA polymerase) – ORF 303 (aminopeptidase C)	NCLDV – NCLDV	276568-278069 (1501 bp)	~1500 bp
ORF 479 (E3 ubiquitin ligase RING1-like protein) – ORF 480 (protein kinase)	Eukaryotic – NCLDV	418693-419562 (1597 bp)	~1600 bp
ORF 019 (Lon protease) – ORF 020 (5’ – 3’ exoribonuclease)	Bacterial – NCLDV	15167-16745 (1578 bp)	~1600 bp

*NCLDV: Nucleocytoplasmic Large DNA Viruses

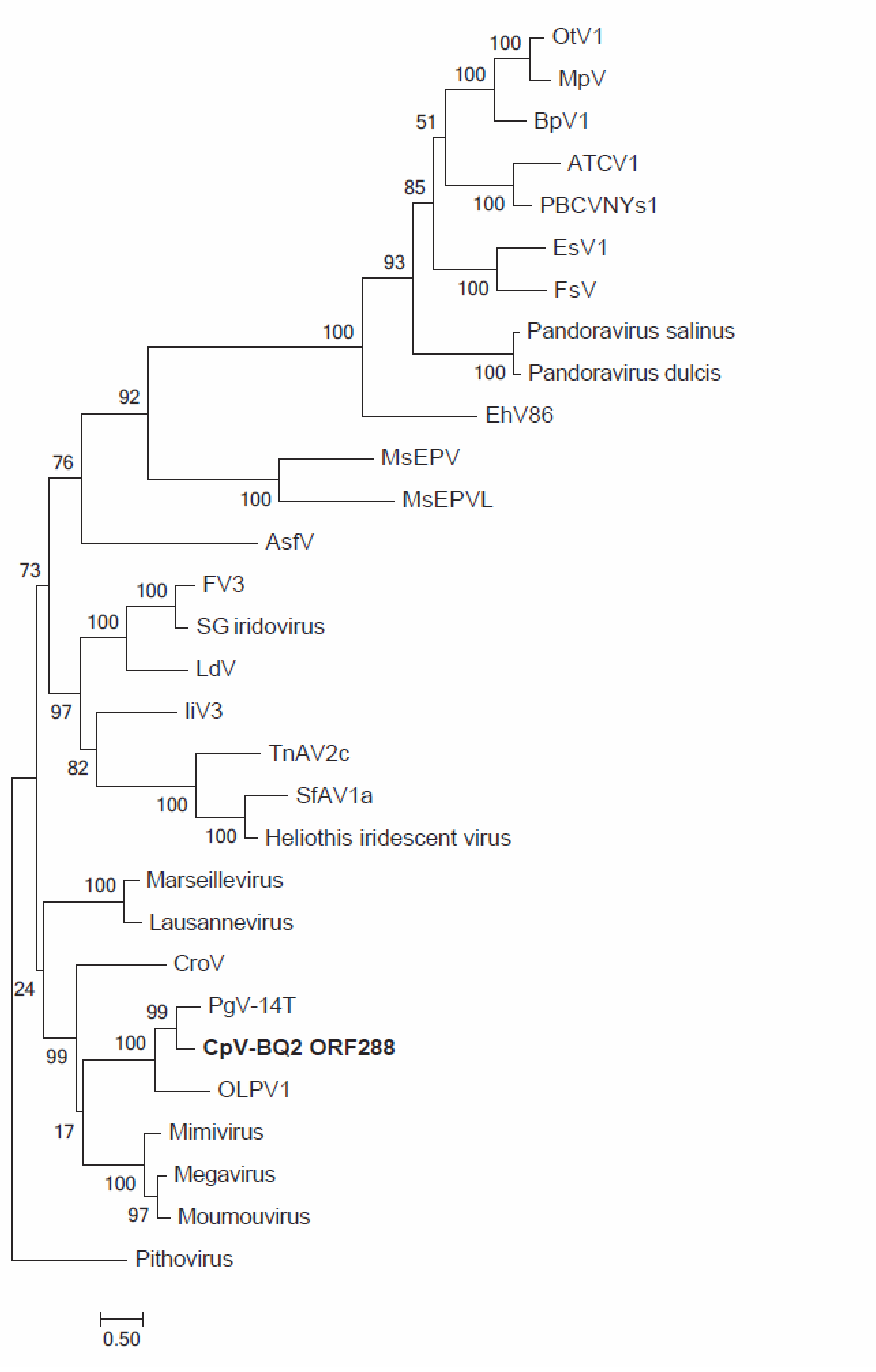

Supplementary Figure 1. Maximum-likelihood phylogenetic tree of the CpV-BQ2 D5 Helicase. Node support (aLRT-SH statistic) >50% are shown.

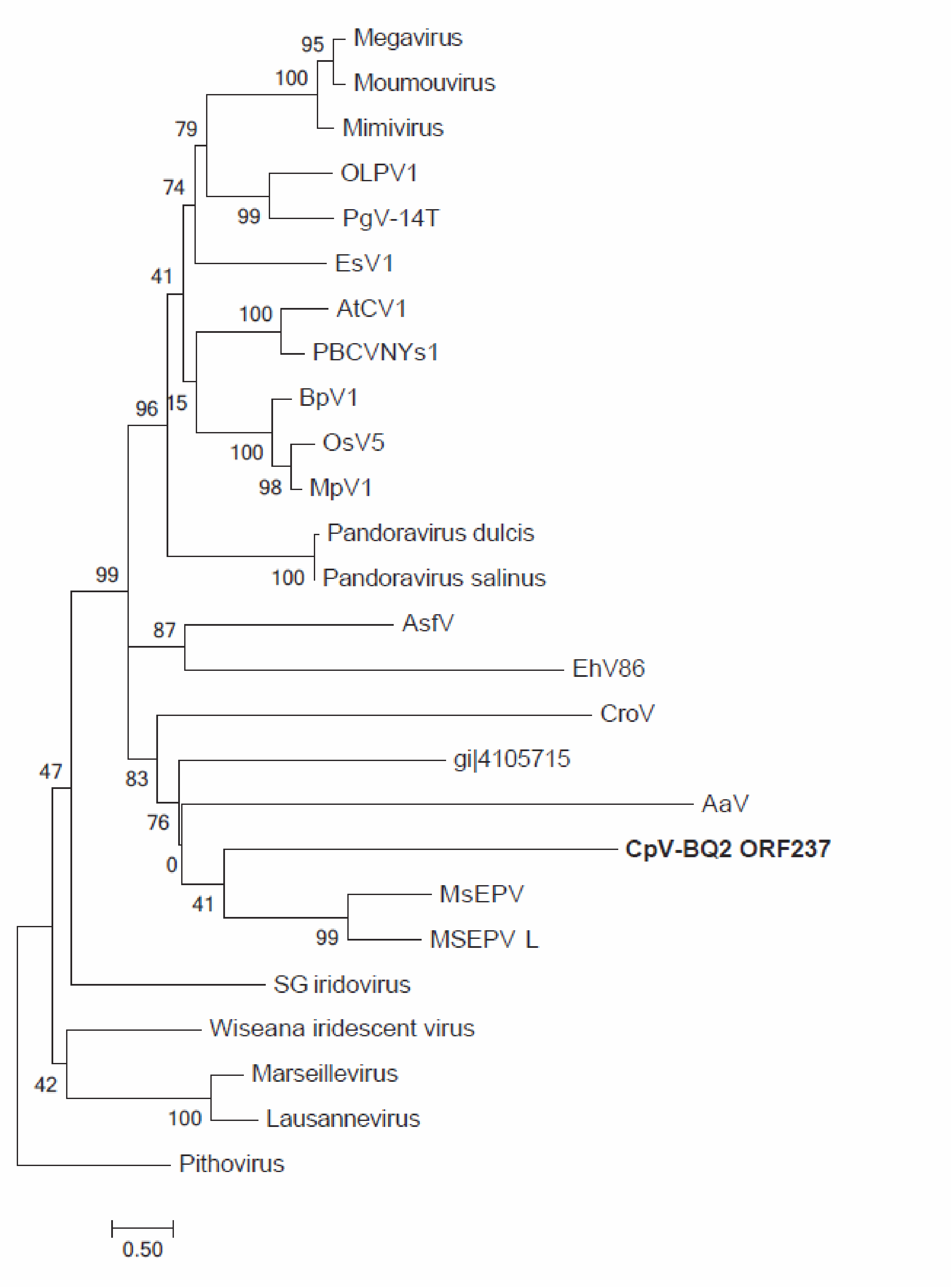

Supplementary Figure 2. Maximum-likelihood phylogenetic tree of the CpV-BQ2 Superfamily II Helicase. Node support (aLRT-SH statistic) >50% are shown.

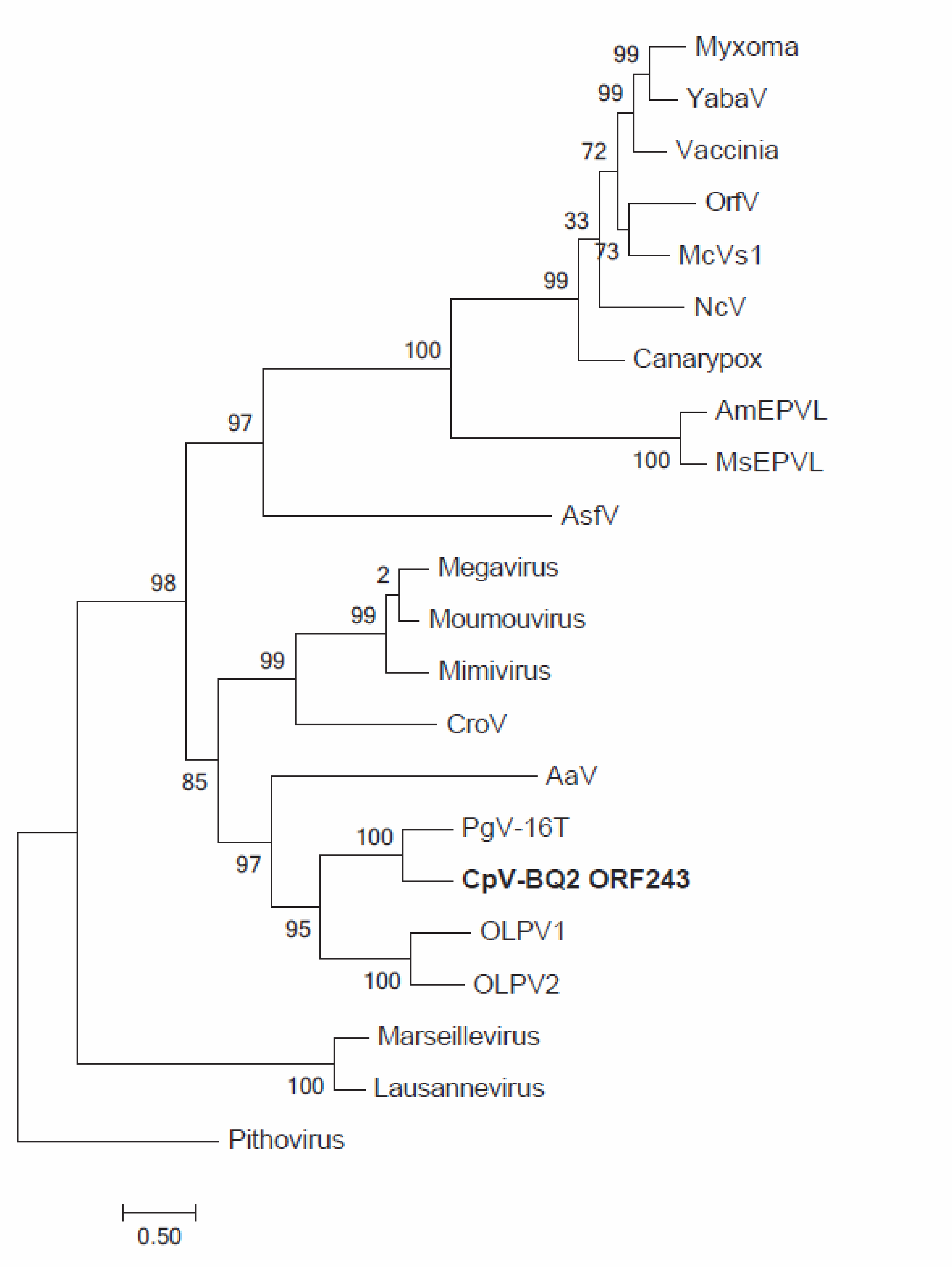

Supplementary Figure 3. Maximum-likelihood phylogenetic tree of the CpV-BQ2 mRNA capping enzyme. Node support (aLRT-SH statistic) >50% are shown.

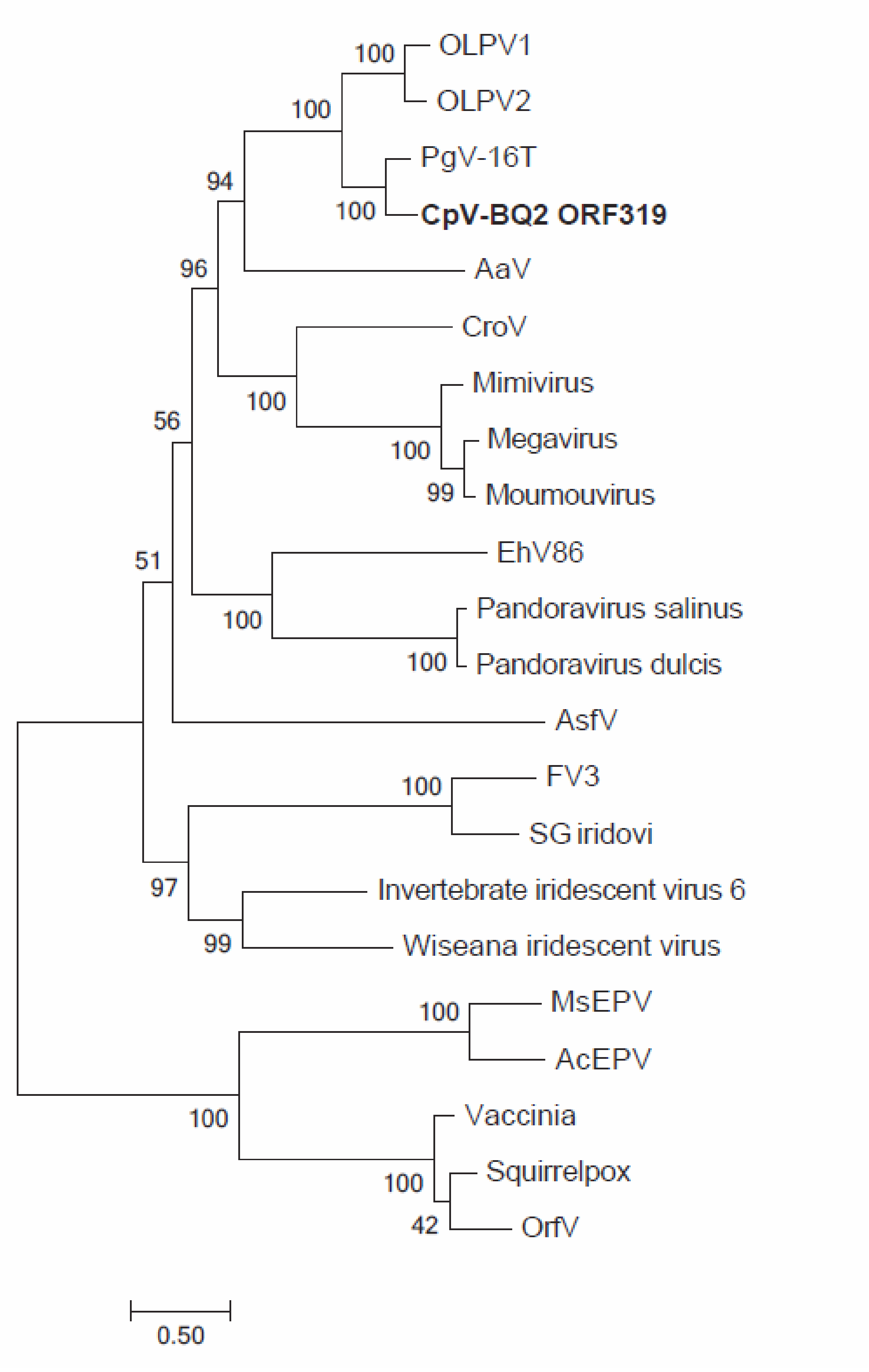

Supplementary Figure 4. Maximum-likelihood phylogenetic trees of the CpV-BQ2 RNA Polymerase α-subunit. Node support (aLRT-SH statistic) >50% are shown.

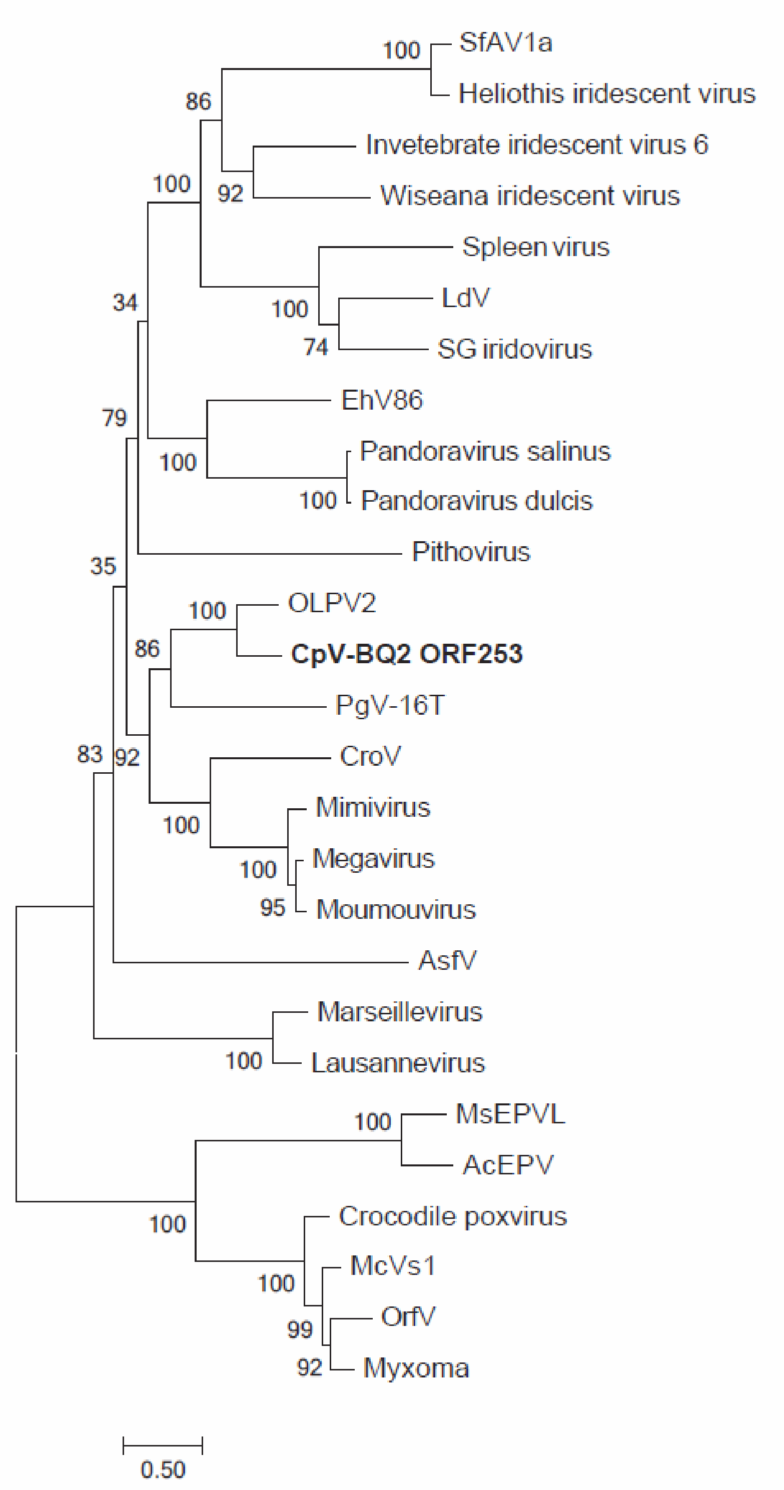

Supplementary Figure 5. Maximum-likelihood phylogenetic trees of the CpV-BQ2 RNA polymerase β-subunit. Node support (aLRT-SH statistic) >50% are shown.

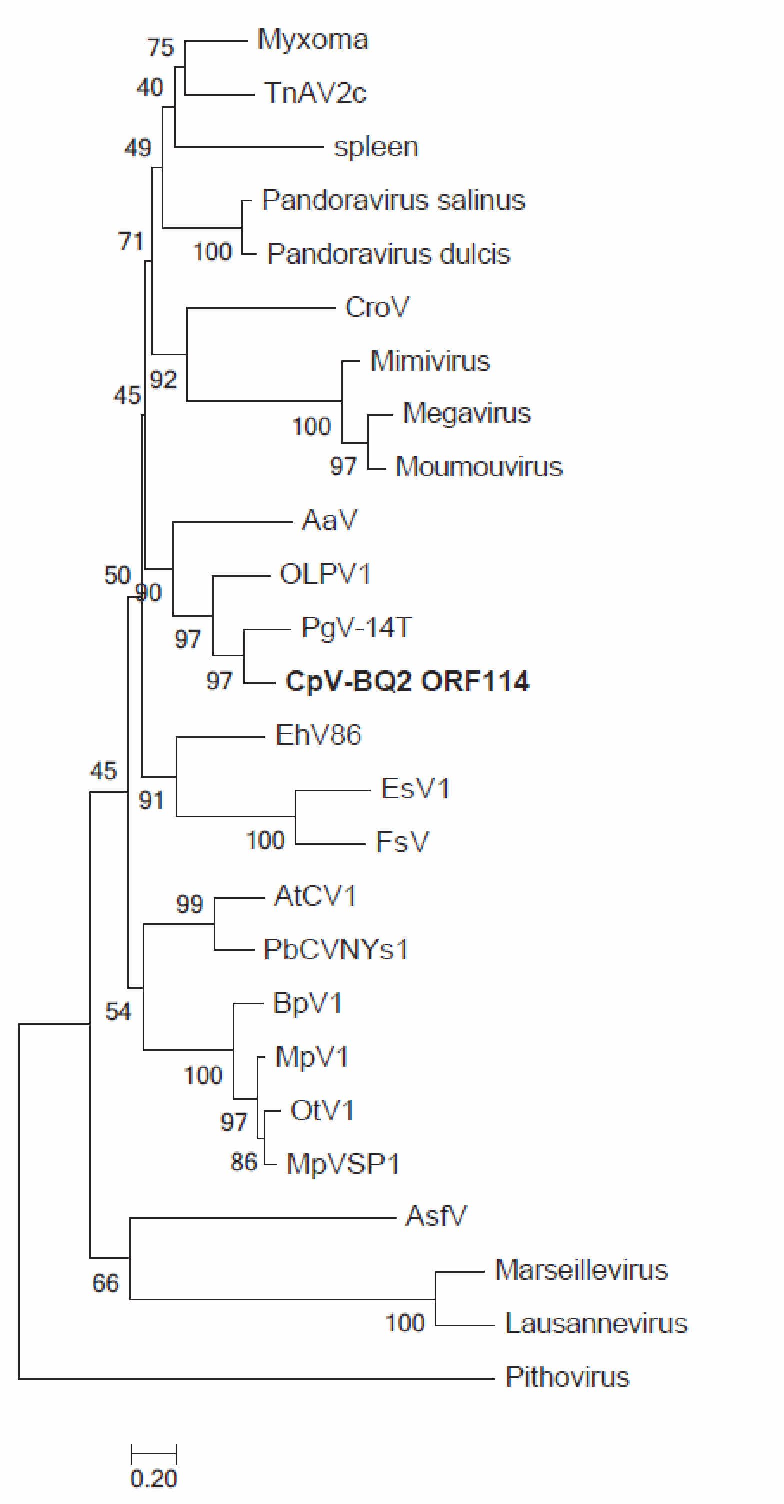

Supplementary Figure 6. Maximum-likelihood phylogenetic trees of the CpV-BQ2 ribonucleotide reductase. Node support (aLRT-SH statistic) >50% are shown.

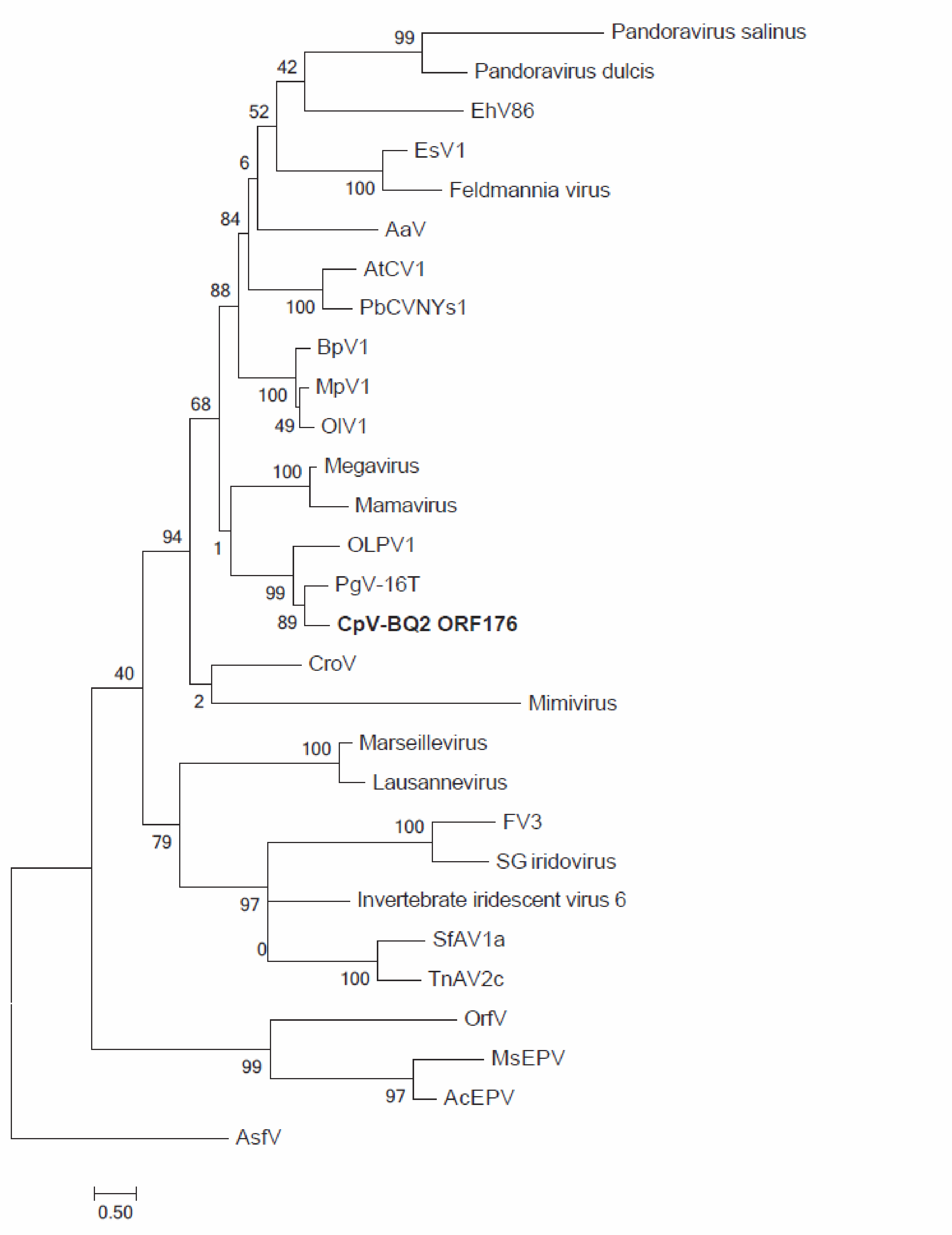

Supplementary Figure 7. Maximum-likelihood phylogenetic trees of the CpV-BQ2 VLTF transcription factor. Node support (aLRT-SH statistic) >50% are shown.
